# Lingual Osseous Choristoma: Case report and literature review of this rare entity

**DOI:** 10.4317/jced.59225

**Published:** 2023-01-01

**Authors:** Ioannis Gkouveris, Nikiforos Kapranos, Georgios G. Mitrou

**Affiliations:** 1 DDS, MSc, PhD. Division of Diagnostic and Surgical Sciences, UCLA School of Dentistry, Los Angeles, CA 90095, USA; 2 DDS, MSc, PhD. Private practice, Athens, Greece; 3 MD, PhD. Laboratory for Molecular Histopathology, Athens, Greece

## Abstract

Intraoral osseous choristoma represents a benign lesion of growing ectopic bone in the soft tissues of the oral cavity. It is considered as rare entity while fewer than 100 cases have ever been reported worldwide. Nevertheless, the pathogenetic mechanism that drives this abnormal ossification still remains controversial. In the present study a case of lingual osseous choristoma in a 50-year old male is presented. The patient was treated with surgical excision and no recurrence was observed. In addition epidemiology, clinical presentation, and pathogenesis are reviewed, serving as a reminder of this rare pathology.

** Key words:**Osseous choristoma, osteoma, tongue.

## Introduction

Choristoma is a tumor-like growth of histologically normal tissue in an ectopic body site (1,2). Those features distinguish choristoma from hamartoma that represents a benign mass of disorganized tissue and teratoma, which is a benign or malignant tumor that originates from germ cells and consists of different types of tissue such as skin, hair, or muscle. Choristoma may be derived from various tissues including bone, cartilage, salivary and sebaceous glands, or muscle and is consequently named according to the tissue of origin (1). Oral choristomas are infrequent and mostly of bone or cartilage origin (2). Oral osseous choristomas are very rare with less than 100 cases reported in the English literature (3). They were initially described as soft tissue osteomas in parallel with the benign bone neoplasm (2,3), but Krolls *et al*. in 1971 introduced the term “osseous choristoma” (4). Their etiopathogenesis remains obscure, and developmental or reactive/post-traumatic origin is mostly considered (1,5).

We present a case of a lingual osseous choristoma and review epidemiology, clinical presentation, and pathogenesis of oral osseous choristomas.

## Case Report

A 50 year-old man presented for evaluation of a mass on the tongue that he noticed more than a year ago, while brushing his teeth. He reported slow enlargement during the last 4 months, causing a “lump in the throat” sensation, without dysphagia, odynophagia, or bleeding. His medical history was noncontributory; he smoked 20 cigarettes per day. No history of intraoral trauma was elicited.

**Figure 1:**
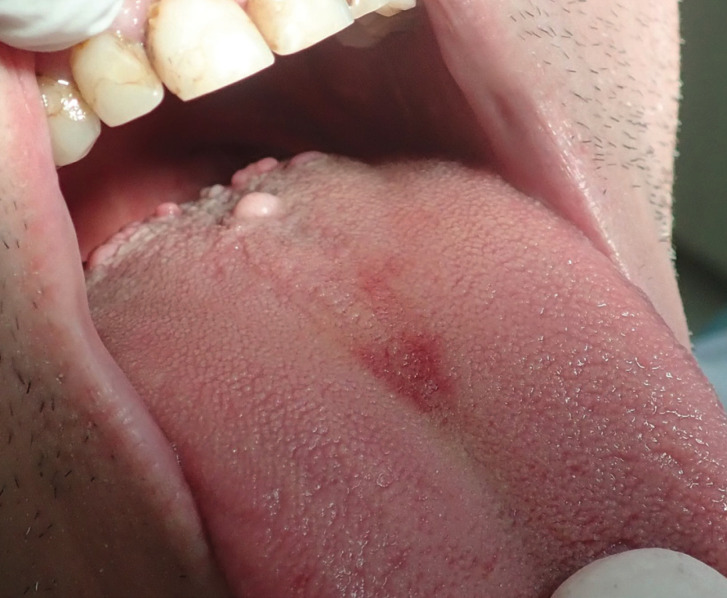
Clinical examination showed a pedunculated, dome-shaped nodule on the distal dorsal tongue, located to the midline. It had pale color, smooth surface, hard consistency and measured approximately 8 mm.

Clinical examination showed a pedunculated, dome-shaped tumor on the distal dorsal tongue, located and close to the midline. It had pale color, smooth surface, hard consistency and measured approximately 8 mm (Fig. 1). The rest of the oral and head and neck examination was within normal limits and no cervical lymphadenopathy was found. With the clinical diagnosis of a benign soft tissue tumor, surgical excision under local infiltration anesthesia was performed. The specimen was fixed in 10% buffered formalin.

**Figure 2:**
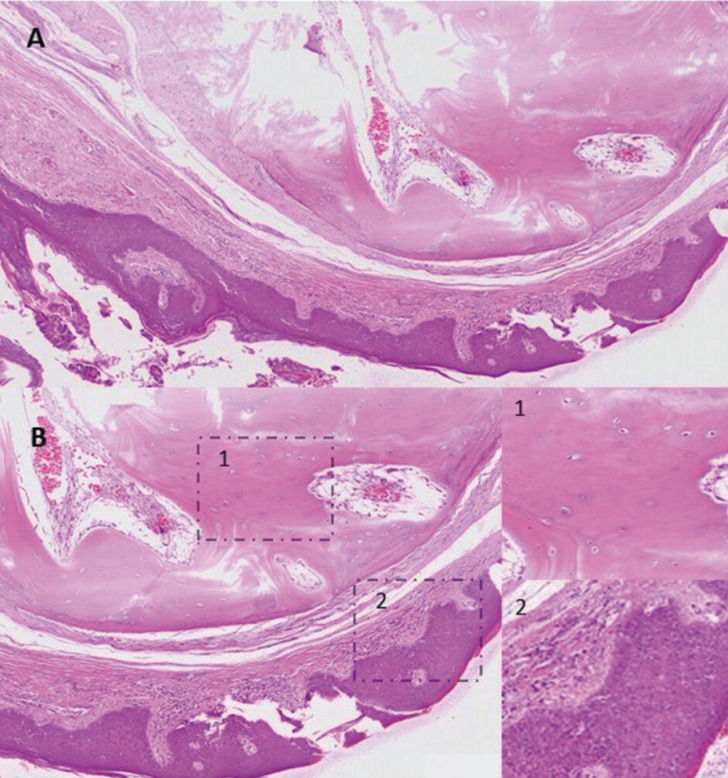
Representative H&E stained section at 5X (A) and 10X (B) magnification demonstrating dense, lamellar bone, with small and irregular marrow spaces containing loose fibrous connective tissue (B, B1 20X), covered by normal-appearing stratified squamous epithelium (B, B2 20X).

Microscopic examination of 5μm thick sections showed that the tumor was composed of dense, lamellar bone, with small and irregular marrow spaces containing loose fibrous connective tissue. It was covered by normal-appearing stratified squamous epithelium (Fig. 2). The diagnosis was lingual osseous choristoma.

Healing was uneventful and there is no evidence of recurrence 12 months after excision.

## Discussion

The case presented herein had a microscopic appearance diagnostic of an oral osseous choristoma. The tongue is the most common location, with 74 cases including the present one reported so far, followed by the buccal mucosa with 15 cases (1) and the soft palate with 2 cases (6,7); one case each have been described in the labial mucosa (1), retro-molar pad (8), submental region (9), masseter muscle (10) and submandibular region (11).

Lingual osseous choristoma shows a strong female predilection, with the female/male ratio being approximately 2.75:1 (2,3). The patients’ age ranges from 5 to 89 years, with most cases occurring in the third decade of life (3). The posterior third of the dorsal tongue, foramen cecum, and circumvallate papillae represent approximately 90% of the cases, with the remaining 10% seen on the middle third of tongue (3). Clinically, it presents as a sessile or pedunculated mass, measuring 0.5 to 2.5 cm, covered by normal appearing mucosa (2). Most cases are asymptomatic, but some patients have reported symptoms, including dysphagia, gagging sensation, pain, vomiting reflex, and nausea (2,3). In contrast to lingual osseous choristomas, their buccal counterparts show only a slight female predilection, with the female/male ratio being 4:3, and the mean patients’ age is 45 years (12). The lesions measured 0.5 to 3 cm; 57% of cases were located in the cheek mucosa, 29% in the bucco-alveolar sulcus and the remaining 14% in the retro-molar area (12). The case presented herein was unusual as it arose in a middle-age man, but showed the typical distal-midline location.

Clinical differential diagnosis of oral osseous choristomas includes mostly benign tumors or tumor-like lesions i.e., fibroma, salivary gland tumors, lipoma, neural tumors, as well as soft tissue cysts (8,9,12). Depending on the extent of calcification, osteolipoma, calcified lymph node, hamartomas or teratomas (10,12), and oral soft tissue calcifications due to hyperparathyroidism may also be considered. Imaging is not suggested as a routine diagnostic procedure, although CT may facilitate diagnosis (5). Definite diagnosis is achieved following biopsy and histological examination.

Considering pathogenesis, developmental and reactive origin have been proposed. Tongue is formed by the fusion of its posterior third, deriving from the third branchial arch, with the anterior two-thirds, deriving form the first branchial arch. Those branchial arches give rise to several normal osseous structures such as incus, malleus, styloid process, and the hyoid bone, through endochondral ossification (3,5). According to developmental theory, oral choristomas originate from brachial arches I, II, and III, trapped in the facial region. Therefore, entrapment of branchial arch derivatives that consequently undergo ossification sounds appealing, as most lesions are located at the midline (1,5). However, it fails to explain the full spectrum of lingual or other intraoral osseous choristomas, as well as the female predilection (1,5). Ossification of lingual thyroid remnants that are commonly seen in women has also, been proposed, but no thyroid tissue has been documented in osseous choristomas (3). Reactive ossification ensuing through pluripotent or ectopic mesenchymal cells exposed to chronic irritation or trauma (13) may be expected at the anterior half and the lateral borders of the tongue that are vulnerable to local inflammation or chronic trauma due to continuous lingual movement, swallowing and articulation (3,5). However, intraoral osseous choristomas present well-developed lamellar bone that is not consistent with calcification caused by chronic irritation (1,7). A multifactorial etiology seems reasonable, with lesions of the posterior tongue being of developmental origin, and those on the anterior tongue or buccal mucosa of post-traumatic etiology (5). The case presented herein was located at the middle of the posterior third of the dorsal tongue, fitting to the developmental theory, while a persistent traumatic stimulus at this area was not identified.

Surgical excision of intraoral osseous choristomas remains the standard treatment, with laser/CO2 excision suggested as an alternative procedure (2). Two cases of recurrence of intraoral osseous choristomas have been documented (10,14), but there is no report of malignant transformation (1). In the present case, conservative surgical excision was curative.
